# Identification of the ilioinguinal and iliohypogastric nerves during open inguinal hernia repair: a nationwide register-based study

**DOI:** 10.1007/s10029-024-03002-2

**Published:** 2024-03-19

**Authors:** V. B. Moseholm, J. J. Baker, J. Rosenberg

**Affiliations:** Center for Perioperative Optimization, Department of Surgery, Copenhagen University Hospital - Herlev Hospital, Borgmester Ib Juuls Vej 1, 2730 Herlev, Denmark

**Keywords:** Nerve identification, Ilioinguinal nerve, Iliohypogastric nerve, Inguinal hernia, Hernia repair, Lichtenstein

## Abstract

**Background:**

Chronic pain remains prevalent after open inguinal hernia repair and nerve-handling strategies are debated. Some guidelines suggest sparing nerves that are encountered; however, the nerve identification rates are unclear. This study aimed to investigate the nerve identification rates in a register-based nationwide cohort.

**Methods:**

This study was reported according to the RECORD guideline and used prospective, routinely collected data from the Danish Hernia Database, which was linked with the National Patient Registry. We included patients ≥ 18 years old, undergoing Lichtenstein hernia repair with information on nerve handling of the iliohypogastric and ilioinguinal nerves.

**Results:**

We included 30,911 open hernia repairs performed between 2012 and 2022. The ilioinguinal nerve was identified in 73% of the repairs and the iliohypogastric nerve in 66% of repairs. Both nerves were spared in more than 94% of cases where they were identified. Female patient sex, emergency and recurrence surgery, general anesthesia, medial and saddle hernias, and large defect size all result in lower nerve identification rates for both nerves.

**Conclusion:**

The Ilioinguinal nerve was recognized in 73% of cases, while the iliohypogastric nerve was recognized in 66% with almost all identified nerves being spared during surgery. Several pre- and intraoperative factors influenced identification rates of the ilioinguinal and iliohypogastric nerve.

## Introduction

The Lichtenstein technique is a common surgical technique for inguinal hernias [1], but despite the commonness of the operation, 1–18% experience moderate to severe postoperative chronic pain [2]. Some studies have shown that nerve-handling strategies might affect chronic pain, but the optimal handling of nerves remains controversial [3, 4]. The two most commonly encountered nerves during open inguinal hernia repair are the ilioinguinal and iliohypogastric nerves [5]. An international guideline for groin hernia management suggests that the encountered nerves should be spared [1]. It has also been recommended that every nerve encountered in the surgical field should be properly identified [6]. It is, however, unclear how often these common nerves are identified in an everyday clinical setting. Most studies reporting on nerve identification are done in a highly controlled environment [7], where identification rates may be inflated, as they are performed by experts who actively search for the nerves. In Denmark, surgeons of all expertise levels have registered operative details of inguinal hernia repairs in the nationwide database since 1998 [8, 9]. In this database, surgeons have been able to register whether nerves were identified since 2011 and this became mandatory in 2016.

This study aimed to investigate the identification rates of the ilioinguinal and iliohypogastric nerves in Denmark. We also wanted to investigate perioperative factors that could affect nerve identification during surgery.

## Methods

This study was a register-based cohort study based on prospectively collected data from the Danish Hernia Database [8], which was linked to the National Patient Registry [10]. This article was reported according to the *Reporting of Studies Conducted using Observational, Routinely collected health Data* (RECORD) guidelines [11].

The Danish hernia database is a nationwide database, where surgeons since 1998 have prospectively registered data after each inguinal hernia repair [8]. The registration rate is approximately 93% of all inguinal hernia repairs in Denmark and contains patient characteristics and perioperative details [8, 12]. Data are collected from all hospitals in Denmark performing inguinal hernia repair, both public and private. Entries in the Danish Hernia Database are linked to the National Patient Registry via each patient’s unique personal identification number enabling cross-link with other variables [10]. In Denmark, every Danish citizen has a unique personal identification number, which gives them access to the Danish healthcare system [13]. This unique personal identification number is used with every healthcare contact in both the public and private sectors.

Data were extracted on all Lichtenstein repairs from the beginning of the database up until data extraction on the 1st of November 2022. The Lichtenstein technique was chosen, as it is both the recommended, and by far the most frequently used, open procedure for inguinal hernia repair [14, 15]. Registration of nerve identification was not possible when the Danish Hernia Database was first implemented, but it became possible by April 1, 2011, and since 2016 registration of nerve identification has been mandatory. Surgeons are required by Danish law to complete an entry in the registry after each completed inguinal hernia repair.

Our primary outcome was the identification rate of the ilioinguinal and iliohypogastric nerves. Through a preliminary investigation of our data, it became evident that the registration rate of nerve handling in the database was steadily increasing up until it became mandatory in 2016. In 2012, the registration rate exceeded 10% and steadily increased thereafter. We decided to include patients from this year and forward. As such, all patient entries from January 1, 2012, to November 1, 2022, were assessed for eligibility. Our eligibility criteria were as follows: adult patients ≥ 18 years of age, undergoing inguinal hernia repair with the Lichtenstein technique, and data had to be available on whether the ilioinguinal and the iliohypogastric nerves were identified or not. Nerve identification and management was coded in the database as a three-point categorical scale: 1 = nerve seen and spared, 2 = nerve seen and resected, and 3 = nerve not seen. From the database, data were also collected on age, sex, hernia size by *European Hernia Society* (EHS) classification [16], hernia type, surgeon authorization ID, anesthesia type, and whether it was a reoperation or not.

For secondary outcomes, we wanted to investigate if nerves were spared or resected when encountered. We also wanted to investigate the association between preoperative and operative factors in nerve identification rates and changes in the identification rates over time. Since registration of nerve identification was not mandatory between 2012 and 2015, we conducted a sensitivity analysis to assess reporting bias by comparing the identification rates in this period with the whole period (2012–2022).

The study size was determined by the number of entries into the Danish Hernia Database, since all eligible patients were included from January 1st, 2012. Statistical analyses were carried out in SPSS (IBM Corp. IBM SPSS statistics for Windows. Armonk, New York: IPM Corp; 2021. Version 28.0.1.0). Test of normality was done with the Shapiro–Wilk tests and by assessing histograms and Q–Q plots. Identification rates between groups were compared using the Pearson Chi-squared test. Patients were grouped by sex, hernia type, hernia size, anesthesia type, and reoperation vs. first repair. Normally distributed data were reported with means and standard deviations, and non-normally distributed data were reported with median and range. Approval for this study was obtained from the Danish Data Protection Agency and the Danish Clinical Quality Assurance Program (protocol number P-2022-609). According to Danish law, this study did not require ethical committee approval [17].

## Results

**Fig. 1:**
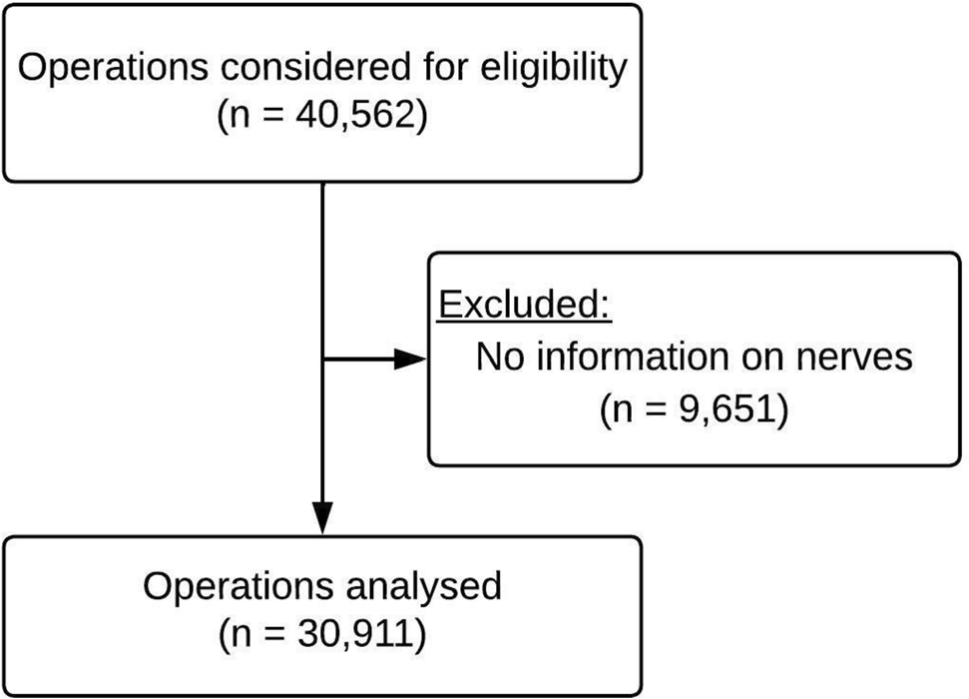
Flowchart describing the inclusion of patients

In total, 30,911 operations were analyzed in this study. A summary flowchart of the inclusion process can be found in Fig. 1. The median age was 68 years (range 18–103), most patients were male (98%), and just over half of the hernias were lateral inguinal hernias. Of the included operations, 94% of repairs were done as elective surgery, with general anesthesia being the dominant form of anesthesia for inguinal hernia repair. Lastly, 9% of surgeries were operations for recurrences, and 86% of these were operated on for their first recurrence. Patient characteristics are summarized in Table 1.

**Table 1:** Patient characteristics

Characteristics 2012–2022	Total (*n* = 30,911)
Demographic	
Male sex, *n* (%)	30,343 (98.2)
Age in years, *median [range]*	68 [18–103]
Surgery type	
Emergency, *n (%)*	1825 (5.9)
Elective, *n (%)*	29,086 (94.1)
Recurrence	
Primary repair, *n (%)*	28,110 (90.9)
Recurrence, *n (%)*	2801 (9.1)
Anesthesia	
General, *n* (%)	24,549 (79.4)
Local, *n* (%)	6020 (19.5)
Spinal, *n* (%)	342 (1.1)
Hernia type	
Lateral, *n* (%)	17,361 (56.2)
Medial, *n* (%)	10,425 (33.7)
Saddle hernia, *n* (%)	2686 (8.7)
Cannot specify, *n* (%)	437 (1.4)
Hernia size	
< 1, *n* (%)	2703 (8.7)
1–2, *n* (%)	17,972 (58.1)
≥ 3, *n* (%)	8535 (27.6)
Unknown, *n* (%)	1701 (5.5)

Hernia size is according to the European Hernia Society Classification [16]

**Fig. 2:**
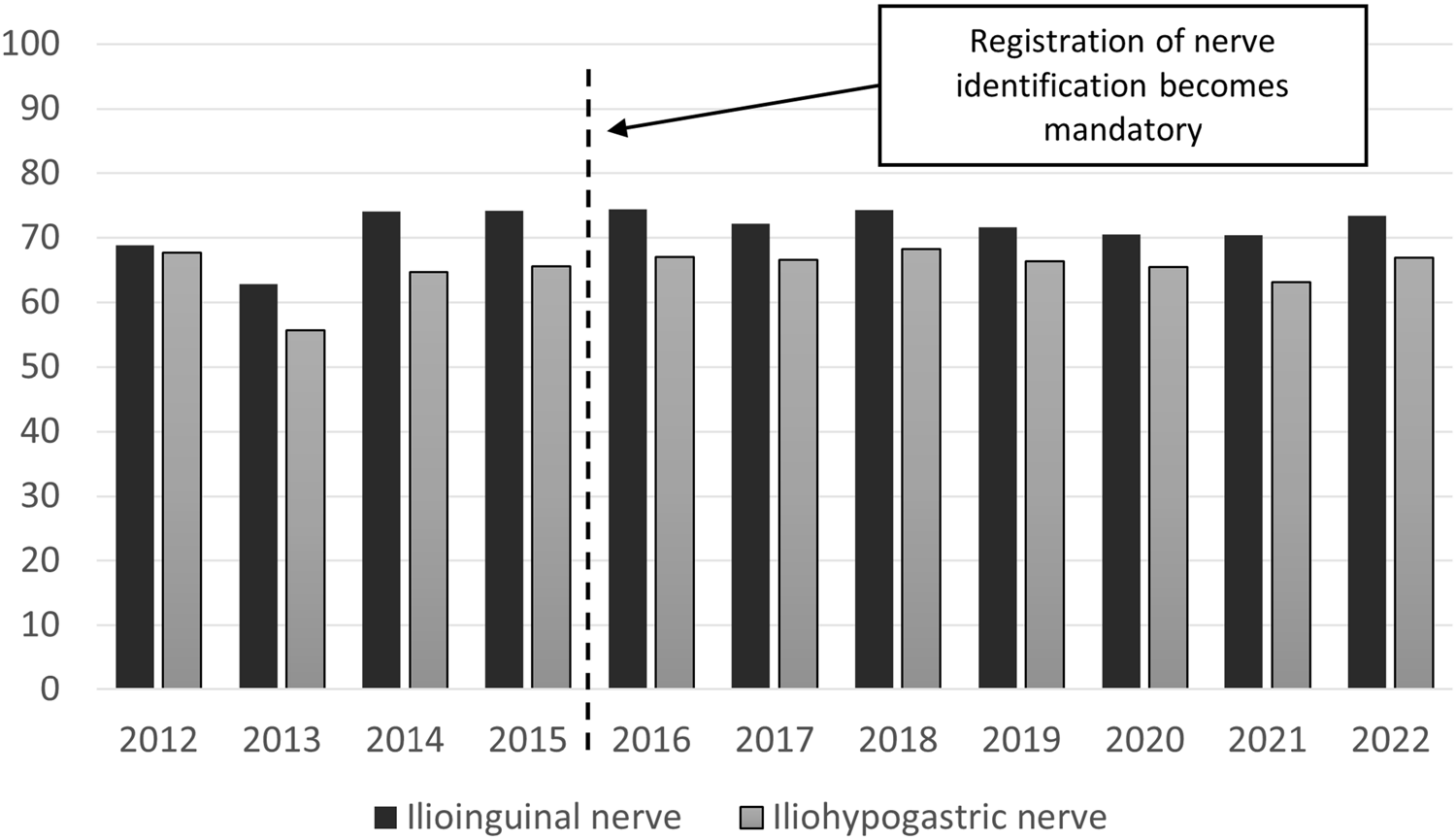
Nerve identification from 2012 to 2022. The dotted vertical line represents the time point from where reporting on nerve identification became mandatory in the Danish Hernia Database

The ilioinguinal nerve was identified in 73% of operations and the iliohypogastric nerve in 66% of the operations (*p* < 0.001). Regarding nerve-handling strategies, a nerve-sparing approach was predominant. When identified, the ilioinguinal nerve was spared in 95% of operations and the iliohypogastric nerve was spared in 96% of operations. Nerve identification over time is summarized in Fig. 2. For the ilioinguinal nerve, sensitivity analysis showed no difference between identification rates before registration of nerve identification became mandatory compared with the whole study period. A significant difference was observed for the iliohypogastric nerve, with an absolute difference of 1.3 percentage points (*p* > 0.001) between the same periods. Thus, nerve identification rates appeared stable, especially after the variable became mandatory in 2016. The outcomes are outlined in Table 2.

**Table 2:** Identification rates and nerve handling

Variable	Total	Ilioinguinal nerve		Iliohypogastric nerve	
	*n*	*n* (%)	*p* value	*n* (%)	*p* value
*Primary outcome*					
Total identifications	30,911	22,454 (72.6)		20,331 (65.8)	
*Secondary outcomes*					
Nerve handling^a^	30,911				
Spared		21,293 (94.8)		19,540 (96.1)	
Resected		1161 (5.2)		791 (3.9)	
Sex			**< 0.001**		**< 0.001**
Female	568	354 (62.3)		317 (55.8)	
Male	30,343	22,100 (72.8)		20,014 (66.0)	
Surgery type			**< 0.001**		**< 0.001**
Emergency	1,850	961 (52.7)		908 (49.8)	
Elective	29,086	21,493 (73.8)		19,423 (66.8)	
Surgery for recurrence			**< 0.001**		**< 0.001**
First time	28,110	20,860 (74.2)		18,867 (67.1)	
Recurrence	2801	1594 (56.9)		1464 (52.3)	
Anesthesia type					
General anesthesia	24,549	17,406 (70.9)	ref	15,885 (64.7)	ref
Local anesthesia	6020	4830 (80.2)	**< 0.001**	4230 (70.3)	**< 0.001**
Regional	342	218 (63.7)	**0.004**	216 (63.2)	0.554
Hernia type^b^					
Lateral	17,361	12,813 (73.8)	ref	11,724 (67.5)	ref
Medial	10,425	7564 (72.6)	**0.028**	6782 (65.1)	**< 0.001**
Saddle	2686	1885 (70.2)	**< 0.001**	1651 (61.5)	**< 0.001**
Unknown	437	191 (43.7)	**< 0.001**	174 (39.8)	**< 0.001**
Hernia size^c^					
< 1	2703	2077 (76.8)	ref	1846 (68.3)	ref
1–2	17,972	13,798 (76.8)	0.989	12,430 (69.2)	0.341
≥ 3	8535	5922 (69.4)	**< 0.001**	5464 (64.0)	**< 0.001**
Unknown	1701	657 (38.6)	**< 0.001**	591 (34.7)	**< 0.001**

Bold values are statistically significant at a confidence level of 95%

Missing data on identification and nerve handling in *n* = 9652 and only between 2012 and 2015 Identification rates are shown as percentages in parentheses out of the total entries in the given group

*n* = nerves identified; Ref = reference group

^a^Percentages on nerve handling are calculated as a proportion of the total amount of identified nerves

^b^Two entries did not specify the type of hernia

^c^Hernia sizes are according to the European Hernia Society Classification [16]

Both nerves were more often identified in men than in women (*p* < 0.001), and nerves were significantly less identified during emergency surgery compared with elective surgery (*p* < 0.001). Operations on recurrent inguinal hernia had a significantly lower identification rate for both nerves compared with primary operations, with a difference of 17.3% points for the ilioinguinal nerve and 14.8% points for the iliohypogastric nerve (*p* < 0.001). Inguinal hernia repairs done under local anesthesia had a significantly higher identification rate than repairs done under general anesthesia. However, operations performed under local anesthesia were performed by a group of surgeons who had a higher number of operations registered, compared with those performed under general anesthesia (*p* < 0.001). Medial and saddle hernias had a significantly lower identification rate compared with lateral hernias (*p* < 0.001). Inguinal hernias with a defect size wider than two fingers, according to the EHS classification [16], had a significantly lower identification rate than hernias with smaller defect sizes.

## Discussion

This study found that the ilioinguinal nerve was identified in about three out of four operations, and the iliohypogastric nerve was identified in two out of three operations. These identification rates remained stable from 2012 to 2022, and a nerve-sparing approach was utilized for almost all of the repairs. Identification rates were dependent on various pre- and intraoperative variables: female patient sex, emergency surgery, recurrence surgery, general and regional anesthesia, non-lateral hernias, and large defect sizes all resulted in lower nerve identification rates.

Another register-based study from Sweden also reported on identification rates, and, with a sample size of 23,259 patients, they found an identification rate for the ilioinguinal nerve of 74% and an identification rate for the iliohypogastric nerve of 56% [18]. These rates are therefore comparable to the ones presented in this study largely because of a similar methodology between studies. A recent systematic review with meta-analysis [5] found a higher identification rate for the ilioinguinal nerve (82%), but a similar identification rate for the iliohypogastric nerve (62%), compared with the present study. The identification rates for the ilioinguinal nerve may be inflated in many studies in the literature, as operations in controlled studies are usually conducted by experts, which could explain a higher identification rate. Furthermore, surgeons participating in studies that specifically intend to report on identification rates may pay more attention to encountered nerves during surgery. Thus, the external validity of those identification rates is questionable compared with the present study, where surgeons of all experience levels in daily routine clinical practice were included, thus markedly increasing the external validity.

Danish surgical guidelines recommend a nerve-sparing approach when nerves are encountered, which was reflected in our results [15]. While the difference in the rate of preservation between nerves was statistically significant, it was hardly clinically relevant. There appears to be an upper limit to the identification rate of the ilioinguinal nerve around 80–90% corresponding well with clinical experience of anatomical variations, as a pooled analysis of 12 anatomic studies found the ilioinguinal nerve present in 84% of dissections [1].

We also found that repairs done under local anesthesia resulted in higher identification rates than under general anesthesia, which may be because they were performed by more experienced surgeons. Hernia defects larger than two fingers according to the EHS classification [16] hindered nerve identification. This may be explained by a large hernia sack in the operative field resulting in anatomical displacement and making nerve identification more difficult during surgery. A previous study supports this, as it showed that the ilioinguinal nerve was identified less in hernia repairs with a defect larger than 3 cm [19]. Both nerves were identified less in women and during emergency surgery. The low identification rate in women may be due to the majority of inguinal hernia repairs being done in men and surgeons might be more used to the male anatomy in the inguinal region. Regarding emergency surgery, the acute nature of emergency surgery may explain why nerves are identified less in these situations. However, proper nerve identification in either case should still be possible.

This study has several strengths. This study was reported according to the RECORD guideline [11]. It was also the largest nationwide cohort study reporting on nerve identification in open inguinal hernia repairs in an everyday surgical setting and thus it ensures a high external validity. The data presented in this study were prospectively collected on a national level, which makes the reported identification rates reliable. This was corroborated by the Danish Hernia Database registration rate of 93% [12]. Furthermore, Danish patient’s unique social security number increases the accuracy of records regarding recurrence and data duplication [13]. This study was limited by not including the genitofemoral nerve, since the genitofemoral nerve is not registered in the Danish hernia database [9]. However, some have argued that the genitofemoral nerve is located too profoundly in the operative field, making routine identification of the genitofemoral nerve a risk of causing iatrogenic nerve injuries during surgery [20]. Nerve identification in the Danish Hernia Database became mandatory in 2016, resulting in missing data before this time, which is a limitation. However, sensitivity analysis showed that mandatory registration did not affect identification rates for the ilioinguinal nerve and, while statistically significant, the difference for the iliohypogastric nerve was clinically negligible.

As the recurrence rates dropped after introduction of mesh repair for inguinal hernia, the significant incidents of chronic groin pain have become increasingly more important. Although no consensus exists, previous studies have advocated for routine nerve identification [4, 21] while others have not been able to find an association between identification of nerves and pain [22, 23]. This study provides valuable insight into the factors that may influence proper nerve identification during open inguinal hernia repair. Furthermore, our results may help form guidelines and future studies on nerve identification and possibly assist in determining the association between nerve identification and chronic pain and other patient-reported outcomes. Future studies should also investigate anatomic variations to assess whether this could be the reason why the nerves are not identified in all repairs. Additionally, it would be valuable to conduct detailed investigations into how the surgeon's volume of inguinal repairs impacts nerve identification rates during these procedures.

## Conclusion

This study found that the ilioinguinal nerve was identified in 73% of the repairs and the iliohypogastric nerve was identified in 66%. Nerves were spared in almost all repairs where they were identified. Female patient sex, emergency and recurrence surgery, general anesthesia, medial and saddle hernias, and large defect sizes all seemed to reduce identification rates of the ilioinguinal and iliohypogastric nerves.

## Data Availability

Data not available due to legal restrictions.
